# Flight Muscle and Wing Mechanical Properties are Involved in Flightlessness of the Domestic Silkmoth, *Bombyx mori*

**DOI:** 10.3390/insects11040220

**Published:** 2020-04-02

**Authors:** Kunpeng Lu, Shubo Liang, Minjin Han, Chunman Wu, Jiangbo Song, Chunlin Li, Songyuan Wu, Songzhen He, Jianyu Ren, Hai Hu, Jianghong Shen, Xiaoling Tong, Fangyin Dai

**Affiliations:** State Key Laboratory of Silkworm Genome Biology; Key Laboratory of Sericultural Biology and Genetic Breeding, Ministry of Agriculture and Rural Affairs; College of Biotechnology, Southwest University, Chongqing 400715, China

**Keywords:** silkworm, flightlessness, domestication, morphology

## Abstract

Flight loss has occurred in many winged insect taxa. The flightless silkmoth *Bombyx mori*, is domesticated from the wild silkmoth, *Bombyx mandarina*, which can fly. In this paper, we studied morphological characteristics attributed to flightlessness in silkmoths. Three domestic flightless *B. mori* strains and one *B. mandarina* population were used to compare morphological components of the flight apparatus, including wing characteristics (shape, forewing area, loading, and stiffness), flight muscle (weight, ratio, and microscopic detail) and body mass. Compared with *B. mandarina*, *B. mori* strains have a larger body, greater wing loading, more flexible wings and a lower flight muscle ratio. The arrangement in microscopy of dorsal longitudinal flight muscles (DLFMs) of *B. mori* was irregular. Comparative analysis of the sexes suggests that degeneration of flight muscles and reduction of wing mechanical properties (stiffness) are associated with silkmoth flightlessness. The findings provide important clues for further research of the molecular mechanisms of *B. mori* flight loss.

## 1. Introduction

Insects occur worldwide and their distributions have been shaped by their ability to fly. Insect flight may have evolved more than 400 million years ago [1]. Flight plays a crucial role in mating, reproduction, finding food, and escaping from predation. However, many winged insects have secondarily become flightless during their evolution [2,3]. In 1854, Wollaston documented that 200 of 550 beetles in the Madeiran archipelago had lost their ability to fly [4,5]. Some orders, such as Grylloblattodea and Siphonaptera, are entirely flightless [6]. In walking sticks, grasshoppers, earwigs, caddisflies, and scorpionflies, flightlessness is common [3,6]. Roff estimated that 5% of pterygotes are flightless [6]. In Lepidoptera species, the flight ability of migratory and non-migratory populations of the monarch butterfly is different [7].

Wings and flight muscles are crucial in insect flight. Flight muscle is the power engine of flying insects, and wings generate the aerodynamic forces required for flight. In many flightless insect species, the flight apparatus has been altered. The flight muscle of flightless grasshopper, *Barytettix psolus* is reduced compared to locust, *Schistocerca gregaria*, which capable of flight. The hemithorax of *Schistocerca gregaria* is filled with large, heavily tracheolated muscles, while it is almost empty in flightless *Barytettix psolus* [8]. In winter moth, *Nyssiodes lefuarius,* the flight muscles of flightless females were strongly reduced in contrast to males capable of flight (some of the flight muscles in pterothorax, mesothorax and metathorax are absent in females) [9]. In 49 species of Lepidoptera, Odonata, Hymenoptera, Coleoptera, Hemiptera, Diptera, and Orthoptera, there was a higher flight muscle ratio associated with greater flight ability [10].

Some flightless insects such as fleas, lice, and walking sticks are typically wingless or only partially winged [3,6]. In some species, such as aphids and planthoppers, the flight-capable insects are macropterous and flightless insects are brachypterous or apterous [11]. Wing shape significantly affects insect flight ability [12,13]. For example, in monarch butterflies, elongated wings are more prevalent in migratory rather than in non-migration populations [7]. Insect wings are flexible and deform significantly under inertial and aerodynamic forces during flight [14,15,16]. The mechanical properties of wings determine how they will change shape in response to aerodynamic forces. The deformation of honeybee wings is related to their mechanical properties [17]. In bumblebees, artificially stiffened wings produced lower maximum vertical aerodynamic force [18]. Compared with a rigid wing, a flexible wing can increase the aerodynamic force in hawkmoths [15]. In contrast, a robotic insect experiment showed that the aerodynamic forces decreased monotonically as the flexibility of the wings increased [19]. Additionally, in neotropical butterflies, body size and wing loading (body mass/wing area) were positively correlated with flight speed [20].

During domestication, some birds and insects have lost the ability to fly. For example, some of the domestic geese, chickens, and silkmoths are flightless [21,22,23]. The domestic silkmoth, *Bombyx mori*, is the only insect that has been entirely domesticated by human beings. *B. mori* was initially domesticated from the wild silkmoth, *B. mandarina*, about 5000 years ago for silk production [24]. Domestication altered several morphologies of *B. mori*. Body color, body size, and cocoon size differ between wild and domestic silkmoths [25,26]. Selection for flightlessness was a key step in domestication success and enabled controlled breeding of *B. mori*. The modification of insect flight apparatus affects their flight ability. In domestication and selective breeding, some organs can be weakened or enhanced. Whether these changes attributed to flightlessness of silkmoth? In this study, we attempted to interpret the morphological reasons for silkmoth flight loss.

We examined morphological variations of body mass, thorax mass, forewing areas, wing shape, wing loading, wing mechanical properties, and flight muscle ratio and compared the measurements between several *B. mori* lines (J106, 872, Dazao) and *B. mandarina*. For body weight, wing loading, and flight muscle ratio, we also compared wild female silkmoths and domestic male silkmoths.

## 2. Materials and Methods

### 2.1. Sample Collection and Rearing

*B. mandarina* larvae were collected from a mulberry orchard (Beibei, Chongqing, China) and reared to establish a colony. The three domestic *B. mori* strains were J106, 872 and Dazao. In a previous silkworm study, the three flightless strains (marked as D04 (J106), D03 (872), and P50-ref (Dazao) in ref. article) and wild silkworms (marked as W11 in ref. article) separated into four distinct clades on a phylogenetic tree [23]. J106 is a landrace, 872 is a commercial strain used in sericulture, and Dazao is a highly inbred strain used as a control in *B. mori* research. We chose the three strains by considering their genetic background. Wild and domestic silkworms were reared on mulberry leaves, in a laboratory under a 12: 12 (light: dark) photoperiod at 25 °C ± 1 °C (the relative humidity was 70% (± 5%)). Table 1 shows the number of individuals in each group. The use and care of experimental insects complied with all relevant local animal welfare laws, guidelines and policies.

### 2.2. Measuring the Flight Apparatus

#### 2.2.1. Body Mass, Flight Muscle Mass, and Flight Muscle Ratio

Body mass of adults was weighed on an electronic balance (Sartorius BSA223S, Sartorius Group, Goettingen, Germany, sensitivity is 0.001 g) after seven hours of eclosion. After weighing, the thorax was removed using scissors and then weighed on the electronic scale. Since the thorax is mainly composed of flight muscles [27], the thorax mass was treated proximately as flight muscle mass. The flight muscle ratio was estimated by the ratio: thorax mass/body mass.

#### 2.2.2. Wing Shape, Wing Area, Wing Loading and Wing Mechanical Properties

Wings were removed using scissors and photographed with a digital camera. Wing area and wing shape were measured using ImageJ 1.47v software. Since it is likely that forewings play a major role in flight of moths and butterflies in generating aerodynamic forces [7,28], and the hind wings are mainly used to maintain balance, we quantified forewing characteristics only. Wing loading (mg/mm^2^) was defined as the ratio: body mass (mg)/forewing area (mm^2^). Wing shape was estimated with the parameter aspect ratio in ImageJ software according to the major and minor axes (major axes/minor axes).

Wing mechanical properties affect the wing deformation and aerodynamic force production of insects. Wing stiffness is a major characteristic of mechanical properties, and it is usually expressed in terms of storage (elastic) modulus (*E’*) [29]. Storage modulus is a dynamic mechanical analysis parameter that evaluates the recoverable deformation energy of materials. It is also known as elastic modulus (in dynamic mechanical analysis). The *E’* of forewings was measured using Dynamic Mechanical Analyzer (DMA-Q800, TA Instruments, USA). Forewings were removed just before the test to ensure the samples were fresh [30]. We trimmed the same part of wings into 1 cm × 0.5 cm rectangles and fixed the regular film slice between two grips of the instrument. A frequency range of 1–100 Hz was used to determine the storage (elastic) modulus *E’*, in a multi-frequency-strain module at 0.1% strain [30,31]. A lower or higher *E’* indicates relatively more flexible or stiff wings.

### 2.3. Microscopy of Flight Muscle

The thorax of silkmoths was dissected and fully covered in optimum cutting temperature (OCT) compound (SAKURA Tissue-Tek O.C.T. Compound, Torrance, CA, USA), then snap frozen in liquid nitrogen (−196 °C). The embedded thorax was sectioned to 15 um using freezing microtome (HM525 NX, Thermo Scientific, Waltham, MA, USA), and stained with hematoxylin and eosin. Images were taken at 10× magnification with a microscope (DP80, Olympus, Tokyo, Japan). Muscle fiber areas were measured using ImageJ 1.47v software. We counted the total number of myofibers of dorsal longitudinal flight muscles (DLFMs) artificially in drawing software of the windows10 system.

### 2.4. Statistical Analysis

As the four silkworm populations have different genetic backgrounds and distribute in distinct branches of the phylogenetic tree [23], we treat them as four independent populations. We used Analysis of Variance (ANOVA) method (IBM SPSS v. 22) to analyze the effects of domestication on the flight apparatus (body mass, flight muscle mass, flight muscle ratio, wing shape, wing area, wing loading). We firstly used a multi-way ANOVA model (morphology = population + sex + population × sex) to evaluate the effects of population and sex on each of the morphological measures. Further, one-way ANOVA tests were employed to assess population effects on the morphology in males and females, respectively. Tukey HSD post hoc tests were performed for the comparisons of mean values of populations (the level of significance was 0.05). To validate whether a morphology affects the flight of silkmoth, we mainly considered the three pairs comparisons between flying *B. mandarina* and each of the three flightless *B. mori*. Morphological measures that showed significant differences in all of these three pairs of flight-flightlessness comparisons were considered related to the flight of silkmoth. 

Both male and female *B. mori* lost their flight ability, which shows that gender did not play a crucial role in the loss of silkmoth flight ability (it does not rule out an influence). Therefore, without considering the influence of gender, we compared male *B. mori* and female *B. mandarina* (the latter is usually heavier than the former) to validate whether the body mass and body-mass related morphologies (wing loading (body mass/forewing area) and flight muscle ratio (thorax mass/body mass)) are essential for the flightlessness of silkmoths. 

For the area of DLFMs, the mean area, and the total number of myofibers of DLFMs, we used a Student’s *t*-test to determine whether differences were significant between domestic and wild silkmoth (the level of significance was 0.05).

## 3. Results

### 3.1. Population Divergence of Morphological Traits

**Figure 1:**
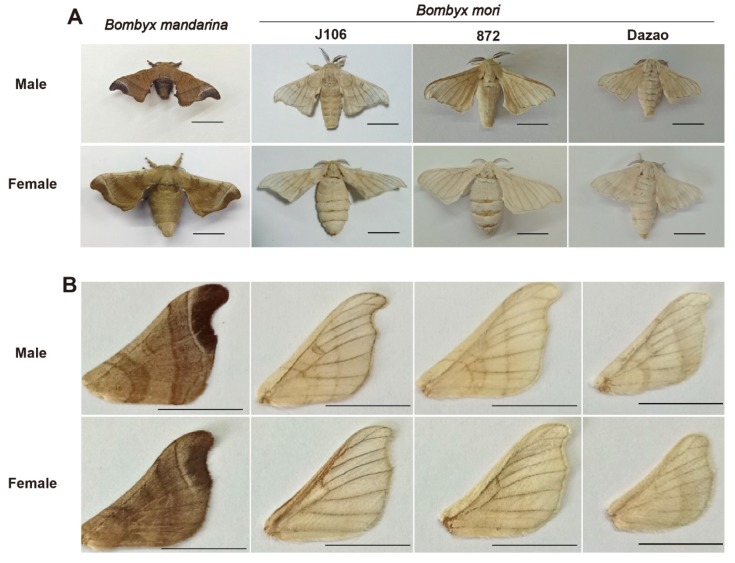
Morphologies of wild and domesticated silkworms and their forewings. (**A**) *B. mandarina* had black body color (left) and the *B. mori* were white (J106, 872, Dazao). (**B**) The color of the forewings was the same as the body color. Several measured characteristics of forewings were listed in Table 1 and plotted in Figure 2 and Figure 3. Bars = 1 cm.

**Figure 2:**
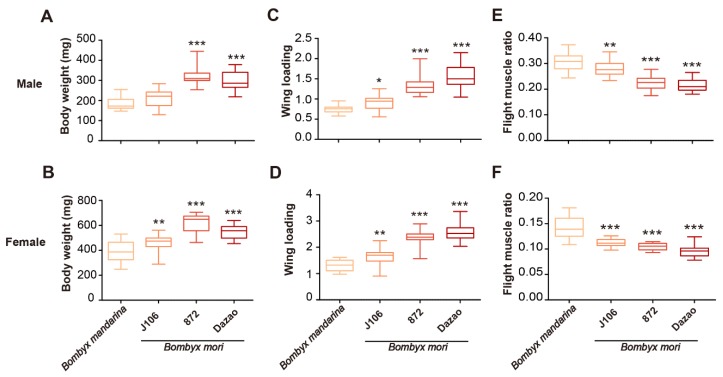
Body weight (mg), wing loading and flight muscle ratio of silkmoths. The five points of box-plot from top to bottom represent the maximum value, the 75th percentile, the 50th percentile (median), the 25th percentile and the minimum value. The box color from light orange to dark red represent *B. mandarina*, J106, 872 and Dazao, respectively. The *X*-axis of the above figures were the same as the bottom. We compared *B. mandarina* to J106, 872, Dazao, respectively using ANOVA. Tukey HSD post hoc tests are shown in Table 3. The significance level is 0.05 (*p < 0.05, **p < 0.01, ***p < 0.001). The body weight (mg) of *B. mori* was larger than that of *B. mandarina* in males (**A**) and females (**B**). The wing loading of *B. mori* was significantly larger than that of *B. mandarina* in males (**C**) and females (**D**). The flight muscle ratio of *B. mori* was significantly lower than that of *B. mandarina* in (**E**) males and (**F**) females.

**Figure 3:**
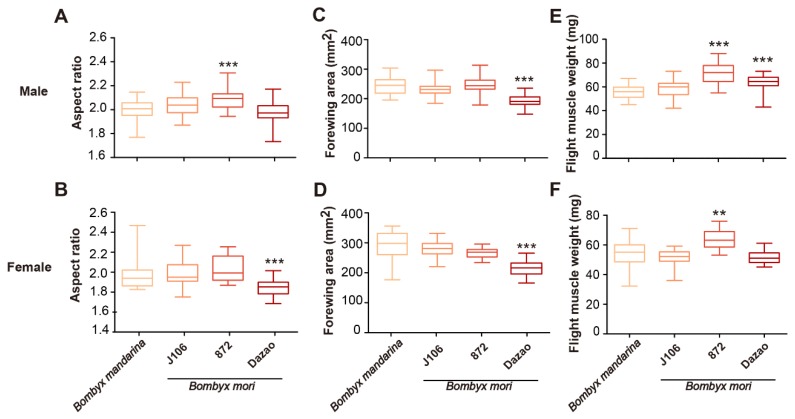
Wing shape (aspect ratio), forewing area (mm^2^) and flight muscle weight (mg) of silkmoths. The box-plot and box color are described in the legend of Figure 2. The *X*-axis of the above figures were the same as the bottom. We applied ANOVA tests between *B. mandarina* and J106, 872, Dazao, respectively. Tukey HSD post hoc tests were shown in Table 3. The significance level is 0.05 (*p < 0.05, **p < 0.01, ***p < 0.001). (**A**) The wing shape (aspect ratio) of *B. mori* was similar to that of *B. mandarina* in males except for 872. (**B**) In females, the wing shape (aspect ratio) of *B. mori* were similar to that of *B. mandarina* except for Dazao. The forewing areas (mm^2^) were similar in males (**C**) and females (**D**) except for Dazao. (**E**) In males, the weight of flight muscles was similar in *B. mandarina* and domestic J106, but the domestic 872 and Dazao had a larger flight muscle weight than *B. mandarina*. (**F**) In females, the flight muscle weight (mg) of *B. mandarina* was similar to J106 and Dazao, and the *B. mori* 872 had heavier flight muscles than *B. mandarina*.

Domestication shaped body type and wing morphology of *B. mori* (Figure 1). Sex and population have significant effects and interactions on measurements of body mass, flight muscle mass, flight muscle ratio, wing shape, wing area and wing loading (Table 2). Comparative results of mean values (Tukey HSD post hoc tests) suggested that body mass, wing loading and flight muscle ratio of *B. mandarina* were significantly different with each of the three *B. mori* populations (except for body mass of J106 males that were similar to *B. mandarina* males) (Table 3; Figure 2). At least one of the three *B. mori* populations showed similarities with *B. mandarina* in the measurements of aspect ratio (wing shape), flight muscle mass and forewing area (Table 3; Figure 3).

### 3.2. Body Mass, Wing Loading, and Wing Mechanical Properties

Body weight of *B. mori* was higher than *B. mandarina* by 1.2–1.8 times (J106: 1.2×; 872: 1.8×; Dazao: 1.6×) in males (Table 1 and Table 3; Figure 2A) and 1.2–1.6 times (J106: 1.2×; 872: 1.6×; Dazao: 1.4×) in females (Table 1 and Table 3; Figure 2B).

**Figure 4:**
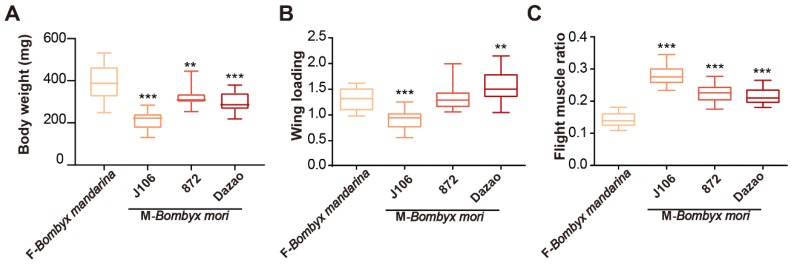
Comparison of body weight (mg), wing loading and flight muscle ratio between female *B. mandarina* (F-wild silkmoth) and male *B. mori* (M-domesticated silkmoth). The box-plot and box color were described in the legend of Figure 2. ANOVA tests were conducted between *B. mandarina* and J106, 872, Dazao, respectively. Tukey HSD post hoc tests are shown in Table 4. The significance level is 0.05 (*p < 0.05, **p < 0.01, ***p < 0.001). (**A**) Body weight (mg) of *B. mori* males was significantly lower than *B. mandarina* females. (**B**) The wing loading of *B. mandarina* females was significantly larger than that of J106 males and similar to that of 872 males. The wing loading of Dazao was significantly larger than that of *B. mandarina* females. (**C**) The flight muscle ratio of *B. mori* males was significantly larger than that of the *B. mandarina* females.

Wing loading of *B. mori* was significantly greater than that of *B. mandarina* (*B. mandarina* < J106 < 872 < Dazao in males and *B. mandarina* < J106 < 872 = Dazao in females; Tukey HSD post hoc tests: males: P*_B. mandarina_*_-J106_ = 0.035, P_872-J106_ < 0.001, P_Dazao-872_ < 0.001; females: P*_B. mandarina_*_-J106_ < 0.001, P_872-J106_ < 0.001, P_Dazao-872_ = 0.163; Figure 2C, D). To further explain whether body mass and wing loading are key factors in determining silkmoth flightlessness, we compared body weight and wing loading of *B. mandarina* females to that of *B. mori* males. The body mass of *B. mandarina* females was significantly larger than *B. mori* males (Table 4; Figure 4A). The wing loading of *B. mandarina* females was larger than J106 and similar to 872 (Table 4; Figure 4B). These results suggested that body weight and wing loading are not key factors of silkmoth flightlessness.

**Figure 5:**
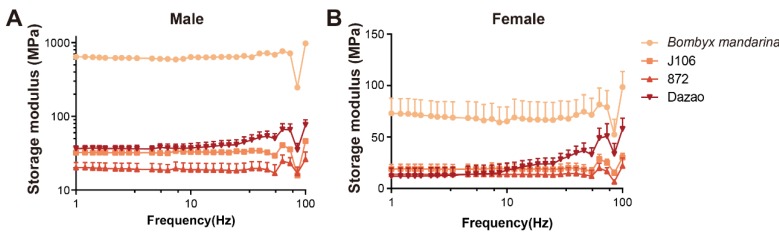
Mechanical properties of forewings. Storage modulus (E’) of forewings of the four populations in males (**A**) and females (**B**). The E’ of *B. mandarina* was higher than that of *B. mori.* Mean + SEM., n = 3 (wild male: n = 2).

The measurements storage modulus (*E’*) were more variable in the high-frequency range, but the *E’* of all three *B. mori* groups floated in the same zone in both sexes (Figure 5A,B). The E’ of *B. mandarina* was always higher than that of *B. mori* (Figure 5A,B), meaning that the wings of *B. mandarina* are stiffer and better able to resist deformation. The stiffer wings of *B. mandarina* maybe have the potential to generate greater lift forces than the softer wings of *B. mori*.

### 3.3. Flight Muscle

We measured flight muscle weight and flight muscle ratio of *B. mori* and *B. mandarina*. Thorax mass was a substitute for flight muscle weight in the study, which was only 1.1–1.3 times (J106: 1.1×; 872: 1.3×; Dazao: 1.1×) greater in *B. mori* males than in *B. mandarina* males (Table 1; Figure 3E). The flight muscle weights were not significant different between J106 and *B. mandarina* (Table 1 and Table 3; Figure 3E). In females, the thorax mass of domestic J106 and Dazao was similar to *B. mandarina* (Table 1 and Table 3; Figure 3F). The greater body mass (Figure 2A,B) and relative invariability of flight muscle weight led to a decreased flight muscle ratio of *B. mori*. The flight muscle ratio corresponded to the trend of *B. mandarina* > J106> 872 = Dazao (Tukey HSD post hoc tests: *p_B. mandarina_*_–J106_ = 0.008, *p*_J106–872_ < 0.001, *p*_872–Dazao_ = 0.8; Figure 2E) in males and *B. mandarina* > J106 = 872 > Dazao in females (Tukey HSD post hoc tests: *p_B. mandarina_*_–J106_ < 0.001, *p*_J106–872_ = 0.157, *p*_872–Dazao_ = 0.021; Figure 2F).

To illustrate whether flight muscle ratio is a key factor in determining silkmoth flightlessness, we compared the flight muscle ratio of *B. mandarina* females to that of *B. mori* males. The results showed that the flight muscle ratio of *B. mandarina* females was significantly lower than that of *B. mori* males (Table 4; Figure 4C), which implied that the ratio is not essential in *B. mori* flight loss.

**Figure 6:**
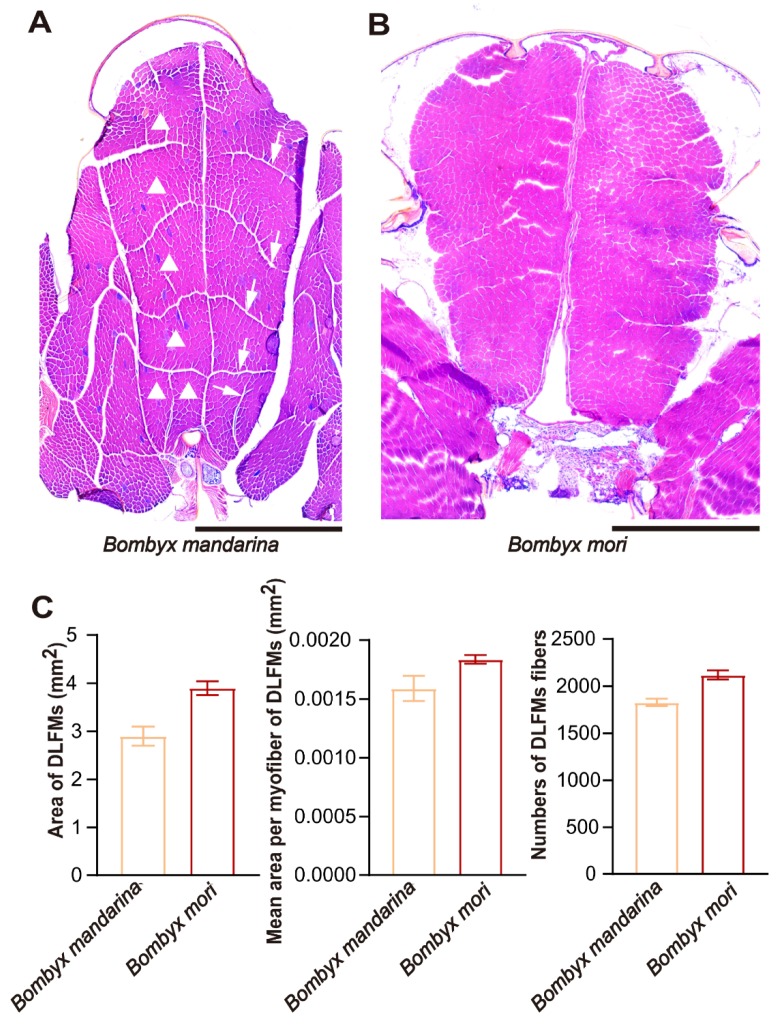
Light microscopy of dorsal longitudinal flight muscles (DFLMs) of silkmoths. (**A**) In *B. mandarina*, there were six DLFMs (triangles) separated by perimysia (arrows) on either side of the midline. All of the DLFMs can be seen in this section, bar = 1 mm. (**B**) In *B. mori*, the DLFMs in each side of the midline are not split into different parts, bar = 1 mm. (**C**) Area of DLFMs (mm^2^), mean area per myofibers in DLFMs (mm^2^) and the number of myofibers in DLFMs were quantified, mean ± SEM., n = 3, Student’s *t*-test, two-tailed, *p*_area_ = 0.127, *p*_mean area_ = 0.370, *p*_number_ = 0.092.

We used microscopy to examine the most prominent muscle class, the dorsal longitudinal flight muscles (DLFMs). In the adult thorax of *B. mandarina*, the DLFMs were composed of two sets of muscle fibers, each set of fibers was separated into six groups of fibers (fascicles) by perimysium (Figure 6A). In *B. mori*, no clearly separated group of fibers was observed in either of the two sets of muscle fibers (Figure 6B). Some perimysia of *B. mori* DLFMs seem to be absent. The area of DLFMs, the mean area and the total number of myofibers of DLFMs were lightly reduced in *B. mori*, but the wild and domestic silkmoths were otherwise similar (Figure 6C. Student’s *t*-test, *p*_area_ = 0.127, *p*_mean area_ = 0.370, *p*_number_ = 0.092). This observation suggested that the arrangement of DLFMs of *B. mori* is irregular. This case is similar to previous observations in flightless hawkmoths [9] and indicates that the irregular DLFMs may have weakened the function of the flight muscle and contributed to *B. mori* flightlessness. 

## 4. Discussion

The evolution of flight has contributed to insect diversification [32]. Flight ability enables insects to disperse, forage and avoid predation. Nevertheless, flightless insects occur in nearly all of the winged orders [5]. Research on flight loss promotes understanding of species adaptation and evolution.

The morphological characteristics differed between *B. mori* and *B. mandarina*, as well as between males and females. Sexual dimorphism is common in insects. For instance, body shape differs between males and females in *Drosophila* [33]. In butterflies and moths, sexual dimorphism occurs often, leading to different body color, body size, body composition (e.g., relative thorax size), wing size and wing shape in males and females [34,35]. Gender also has a significant effect on the morphological characteristics of silkmoths, but flightlessness is not a dimorphic character in *B. mori* since both sexes are flightless.

Our results show that the interaction between population and sex affected flight-related morphologies. The origins (genetic backgrounds) of the *B. mori* used in the study were different, suggesting that they might have experienced different selective pressure during their domestication. For example, the 872 strain is a commercial race and fecundity might be a preferred direction of domestication. The J106 strain is a landrace and easy breeding is more important for them. In these cases, both artificial selection and sexual selection played crucial roles. Throughout the life cycle, females typically allocate more energy for reproduction and males usually allocate more energy for fighting for mating opportunities [34]. In this way, their morphologies would be affected differently. We believe that the effects of sexual and artificial selection on flight-related morphologies is the reason for the interaction between sex and the population. Flight loss of *B. mori* probably occurred under artificial selective pressure rather than sexual selective pressure. Thus, we focused on the morphological differences between *B. mandarina* and *B. mori*.

The morphological features of body type, wings and flight muscles differ between *B. mori* and *B. mandarina*. These include body mass, wing loading, wing mechanical properties, and flight muscle ratio. During domestication, silkworms were selected for greater mass to increase silk production [26]. With increased body mass, the wing loading (body mass/forewing area) of *B. mori* increased. Research on birds and Lepidoptera demonstrated a negative correlation between flight ability and wing loading [20,22]. In butterfly *Pararge aegeria*, acceleration capacity was positively correlated with wing loading and body mass [36]. A high or low wing loading and body mass does not always result in poor flight performance. Flightless domestic silkmoths have larger body mass and wing loading than flying wild silkmoths. However, the flying female *B. mandarina* had a larger body mass and greater wing loading than flightless *B. mori* males. This suggests that wing loading and body mass are not the key factors of silkmoth flight loss.

Flapping wings generate aerodynamic forces in insect flight. The wing shape of flying insects changes considerably in spanwise and chordwise directions [37]. The motion of wings and their three-dimensional shape have a significant effect on lift forces [38,39,40]. In a robotic insect experiment, the aerodynamic forces decreased monotonically as the flexibility of wings increased [19]. In contrast, flexible wings produced larger aerodynamic force than rigid wings in hawkmoths and bumblebees [15,18]. These studies showed that flexibility or stiffness of insect wings does not always indicate enhancement or reduction of aerodynamic forces. Rajabi and Gorb believe that a balance between flexibility and stiffness is needed [41]. The flexibility of wings should be kept in a suitable range. Wings that are too soft cannot resistant aerodynamic forces and excessively rigid wings cannot form dimensional shape. The storage modulus (*E’*) of domestic *B. mori* was lower than *B. mandarina*. *E’* usually reflects the stiffness of materials [29]. The lower *E’* of *B. mori* wings indicated lower stiffness (more flexibility). We suppose that the reduced flexibility of *B. mori* wings compromises the balance between stiffness and flexibility and reduced the capacity to generate lift. This change might have affected the flight ability of *B. mori* and was involved in silkmoth flightlessness.

Wingbeats require considerable energy [42,43,44], which is provided by the flight muscles. Dysfunction of flight muscles can lead to weakened flight ability. For example, the degeneration of flight muscles in *Drosophila* leads to flightlessness or reduced flight ability [45,46,47]. We found that *B. mori* had a reduced proportion of flight muscles. However, in a comparison between female *B. mandarina* and male *B. mori*, female *B. mandarina* had a significantly lower flight muscle ratio. This suggests that the lower flight muscle ratio of *B. mori* was not responsible for flightlessness. The perimysia of *B. mori* flight muscle seems to be absent, which implies a degeneration of this powerful engine. The structure of the perimysium provides an important mechanical function in skeletal muscles [48], such as the transmission of forces, passive elasticity, and stiffness of muscles [48,49,50]. In *Nyssiodes lefuarius* (Lepidoptera: Geometridae), the dorsal longitudinal muscles of flightless females have no clearly separated bundles in contrast to flying males [9]. This situation is similar to the DLFMs of the domestic silkmoth. The degeneration of silkmoth DLFMs might have affected the construction of flight muscles and led to an insufficient energy supply.

To increase silk production, larger silkworms have been selected for breeding. Based on our understanding of silkworm domestication, we believed that excess body weight is the major reason for silkworm flightlessness. However, the view is purely anecdotal and might be misleading further research on silkmoth flightlessness. By measuring and comparing the morphology of the flight apparatus of silkmoths, we demonstrated that body weight, flight muscle ratio, wing loading, and wing mechanical properties were different between wild and domestic silkmoth. They might affect silkmoth flight ability. However, comparisons between flying females and flightless males demonstrated that the body weight, wing loading and flight muscle ratio were not attributed to silkmoth flightlessness. Then, we speculated that flight muscle structure and wing mechanical properties (stiffness) were key aspects in flight loss. 

To date, most studies of the relationship between morphology and the loss of flight have been conducted on insects that have undergone natural selection (in wild field). This study expanded knowledge of natural selection examples to domestic insects (undergoing artificial selection). The findings provide important clues for further research on the molecular mechanisms of *B. mori* flight loss. Morphological data have limitations for explaining complex issues related to loss of flight in species. Additional studies on physiology and molecular biology would increase our understanding of energy metabolism and the molecular mechanism of silkmoth flightlessness. 

## 5. Conclusions

We measured and compared flight apparatus that could influence silkmoth flight ability and verified that flight muscle and wing mechanical properties (stiffness) are essential for silkmoth flightlessness. The measurements are useful for understanding silkmoth flight loss. The result offered a dependable direction for future research in the flight loss of the silkmoth. Despite the findings, further research should be conducted to determine whether the energy supply is sufficient. Genes involved in flight muscle development should be examined in the wild and domestic silkmoth (e.g., expression and nucleotide sequence of genes). 

## Figures and Tables

**Table 1:** Numbers of measured individuals and mean (±SD) values of morphological characteristics. Means were shown separately for males and females. Results of Analysis of Variance (ANOVA) tests of the measurements are shown in Table 2 and Table 3. Abbreviated letters and digital superscript are indicated by footnotes.

Population	Origin	Sex	N	FMR	Wing Loading (mg/mm^2^)	Forewing Area (mm^2^)	Body Mass (mg)	FMM (mg)	Aspect Ratio
**Wild Silkmoth**	**Wild-caught**	M	20	0.31 (±0.03)	0.75 (±0.08)	246.98 (±30.73)	184.35 (±30.97)	55.75 (±6.32)	1.99 (±0.09)
		F	13	0.14 (±0.02)	1.32 (±0.21)	293.47 (±49.23)	385.85 (±85.40)	54.08 (±9.89)	1.98 (±0.17)
**Domestic Silkmoth**	**J106**	M	25	0.28 (±0.03)	0.92 (±0.17)	232.01 (±23.27)	213.76 (±40.83)	58.88 (±7.19)	2.04 (±0.09)
		F	30	0.11 (±0.01)	1.67 (±0.26)	280.38 (±26.40)	464.13 (±59.42)	51.67 (±4.90)	1.99 (±0.11)
	**872**	M	25	0.22 (±0.03)	1.33 (±0.23)	245.41 (±25.99)	322.92 (±43.67)	71.44 (±8.65)	2.09 (±0.08)
		F	17	0.10 (±0.01)	2.36 (±0.38^†^)	267.15 (±17.88^†^)	617.12 (±73.24)	64.35 (±6.61)	2.04 (±0.14^†^)
	**Dazao**	M	32	0.22 (±0.02)	1.56 (±0.28)	192.63 (±19.43)	298.41 (±44.19)	63.81 (±5.93)	1.98 (±0.09)
		F	33	0.09 (±0.01)	2.57 (±0.31)	215.56 (±24.49)	549.24 (±52.26)	51.73 (±4.24)	1.84 (±0.09)

N = numbers of individuals; M = males; F = females; FMR = Flight muscle ratio; FMM = Flight muscle mass; ^†^N = 11.

**Table 2:** Multi-way ANOVA tests of measurements of flight apparatus. The table shows the effects of sex, population and their interaction (population × sex) on morphological measurements. The significance level is 0.05 (Tukey HSD post hoc tests).

Measurements	Factors	Df	F Values	*p* Values
**Body Mass**	Sex	1	903.720	<0.001
	Population	3	80.771	<0.001
	Population × sex	3	4.314	=0.006
**Flight muscle Mass**	Sex	1	50.855	<0.001
	Population	3	35.499	<0.001
	Population × sex	3	4.683	=0.004
**Forewing Area**	Sex	1	68.719	<0.001
	Population	3	62.361	<0.001
	Population × sex	3	3.317	=0.021
**Aspect Ratio (wing shape)**	Sex	1	14.286	<0.001
	Population	3	19.067	<0.001
	Population × sex	3	3.146	=0.026
**Wing Loading**	Sex	1	450.410	<0.001
	Population	3	162.878	<0.001
	Population × sex	3	7.310	<0.001
**Flight Muscle Ratio**	Sex	1	2082.351	<0.001
	Population	3	95.981	<0.001
	Population × sex	3	19.318	<0.001

**Table 3:** One-way ANOVA tests were conducted separately for males and females. The table shows Tukey’s comparison between *B. mandarina* and each of the three *B. mori* populations, respectively. The significance level is 0.05 (Tukey HSD post hoc tests).

Measurements	Males			Females		
	Wild Silkmoth	Domestic Silkmoth	*p* Values	Wild Silkmoth	Domestic Silkmoth	*p* Values
**Body Mass** **(mg)**	Wild silkmoth	J106	=0.085	Wild silkmoth	J106	=0.002
		872	<0.001		872	<0.001
		Dazao	<0.001		Dazao	<0.001
**Flight Muscle Mass** **(mg)**	Wild silkmoth	J106	=0.455	Wild silkmoth	J106	=0.615
		872	<0.001		872	<0.001
		Dazao	=0.001		Dazao	=0.624
**Forewing Area** **(mm^2^)**	Wild silkmoth	J106	=0.182	Wild silkmoth	J106	=0.540
		872	=0.997		872	=0.136
		Dazao	<0.001		Dazao	<0.001
**Aspect Ratio** **(wing shape)**	Wild silkmoth	J106	=0.308	Wild silkmoth	J106	=0.979
		872	=0.004		872	=0.523
		Dazao	=0.920		Dazao	=0.005
**Wing Loading**	Wild silkmoth	J106	=0.035	Wild silkmoth	J106	=0.003
		872	<0.001		872	<0.001
		Dazao	<0.001		Dazao	<0.001
**Flight Muscle Ratio**	Wild silkmoth	J106	=0.008	Wild silkmoth	J106	<0.001
		872	<0.001		872	<0.001
		Dazao	<0.001		Dazao	<0.001

**Table 4:** One-way ANOVA test of wild females and domesticated males. The mean (±SD) values were the same as shown in Table 1. The table shows Tukey’s comparison between *B. mandarina* females and each of the three *B. mori* males, respectively. The significance level is 0.05 (Tukey HSD post hoc tests).

Measurements	Wild Silkmoth	Means (± SD)	Domestic Silkmoth	Means (±SD)	*p* Values
**Body Mass (mg)**	Wild silkmoth (Female)	385.85 (±85.40)	J106 (male)	213.76 (±40.83)	<0.001
			872 (male)	322.92 (±43.67)	=0.003
			Dazao (male)	298.41 (±44.19)	<0.001
**Wing Loading**	Wild silkmoth (Female)	1.32 (±0.21)	J106 (male)	0.92 (±0.17)	<0.001
			872 (male)	1.33 (±0.23)	=0.999
			Dazao (male)	1.56 (±0.28)	<0.010
**Flight Muscle Ratio**	Wild silkmoth (Female)	0.14 (±0.02)	J106 (male)	0.28 (±0.03)	<0.001
			872 (male)	0.22 (±0.03)	<0.001
			Dazao (male)	0.22 (±0.02)	<0.001
